# Disaster Stressors and Psychological Well-Being After a Flood

**DOI:** 10.1093/geroni/igaa057.1979

**Published:** 2020-12-16

**Authors:** Katie E Cherry, Alyssa De Vito, Matthew Calamia, Emily Elliott

**Affiliations:** Louisiana State University, Baton Rouge, Louisiana, United States

## Abstract

Hurricanes and floods have mental health consequences for younger and older adults alike. In August of 2016, historic flooding in Baton Rouge, Louisiana resulted in billions of dollars in damages. In this study, we compared 223 mostly middle-aged and older adults on mental health indicators. The majority of the sample (n = 137) were non-coastal residents and the remainder (n = 86) were former coastal residents who had permanently relocated inland after catastrophic losses in the 2005 Hurricanes Katrina and Rita. Multiple regressions confirmed elevations in symptoms of depression and post-traumatic stress for both adults with flood damage in 2016 and also those who doubly flooded in 2016 and 2005. Age had a protective effect for symptoms of depression and worry. Prior lifetime trauma was a risk factor for depression. Implications of these data for understanding age-related vulnerabilities after multiple disasters are discussed with suggestions to strengthen post-disaster resilience.

